# Vascular Reconstruction With Autologous Venovenous Grafting After Complete Transection of the Superior Mesenteric Vein (SMV) During Right Hemicolectomy (RH) With Complete Mesocolic Excision (CME)

**DOI:** 10.7759/cureus.103771

**Published:** 2026-02-17

**Authors:** Nikolaos Kokoroskos, Nikolaos Dalamagkas, Dimitris Fagkrezos, Dimitrios Staramos, Konstantinos Manes

**Affiliations:** 1 Department of General Surgery, Konstantopouleio-Patision General Hospital of Nea Ionia, Athens, GRC; 2 Department of Computed Tomography, Radiology, Konstantopoulio General Hospital, Nea Ionia, GRC; 3 Department of Vascular Surgery, Konstantopouleio-Patision General Hospital of Nea Ionia, Athens, GRC

**Keywords:** complete mesocolic excision (cme), grafting, right hemicolectomy, smv injury, vascular repair

## Abstract

Right hemicolectomy (RH) with complete mesocolic excision (CME) for right-sided colon cancer has been recommended by experts as the optimal approach in terms of oncologic and survival outcomes. However, intraoperative adverse events, such as iatrogenic vascular injuries, are more commonly associated with CME as opposed to the conventional method. This report delineates a severe injury to the superior mesenteric vein (SMV) with complete transection of the vessel during RH/CME, which was definitively managed with autologous venovenous grafting. Awareness of the risks, familiarity with the anatomy, and adequate training are mandatory for colorectal surgeons in order to master a technique with a steep learning curve and be able to manage potentially disastrous complications.

## Introduction

Right hemicolectomy with complete mesocolic excision (CME) for adenocarcinoma of the right colon, although not universally adopted yet, has been proposed by several authors as the standard of care in terms of oncological outcome and survival rate, particularly for stage III disease [1,2]. Prospective studies across the world are being conducted to investigate factors that might have an impact on the safety of minimally invasive right hemicolectomy (RH) with CME [3]. There is rapidly expanding data endorsing CME over the conventional approach, as well as systematic efforts from expert societies to teach colorectal surgeons to achieve mastery of the technique despite the steep learning curve [4-8]. However, despite the oncologic benefit, CME has been associated with a higher risk of significant vascular injuries around the superior mesenteric vein (SMV) and other organ injuries compared with the traditional operation [9]. SMV transection, although rare, is one of the most severe complications during CME. Prompt identification of the injury pattern and available expertise for repair are paramount. The authors’ intention in this case report was to share their experience regarding the management of this infrequent, yet severe and potentially devastating intraoperative complication.

## Case presentation

We present a case of a 72-year-old male with a BMI of 33 and an unremarkable medical history, who was diagnosed with adenocarcinoma of the hepatic flexure. A diagnostic colonoscopy was undertaken due to the new onset of symptoms, namely, bloating and a change in bowel habits. The endoscopy revealed a right colic flexure tumor that had a circumferential growth and was causing moderate lumen stenosis. The pathology report recognized a moderately differentiated, microsatellite-stable colon adenocarcinoma. The rest of the pertinent staging imaging revealed neither metastatic disease, nor ascites, nor local advancement. No vascular anatomic variations were detected on the preoperative multi-detector CT scan. The patient was cleared for surgery, and a right extended hemicolectomy with complete mesocolic excision was planned.

**Figure 1:**
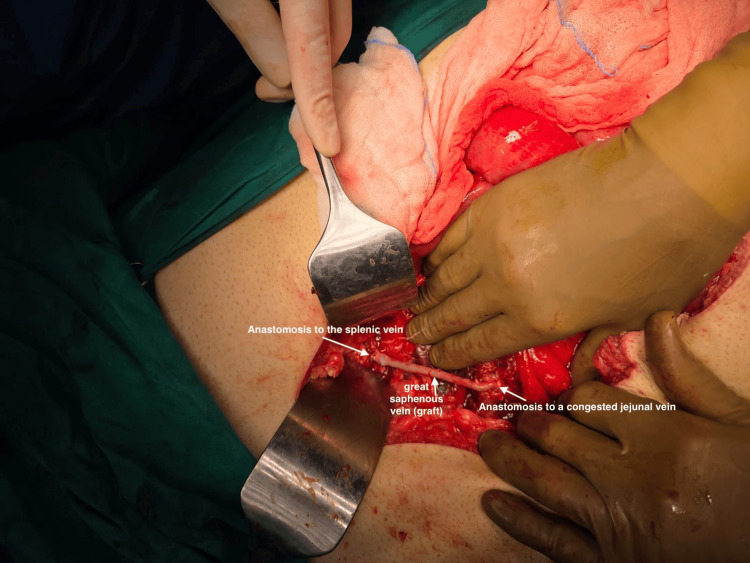
Intraoperative image of the final form of the vascular reconstruction On the right: distal end-to-side anastomosis of the graft with a congested jejunal venous branch. On the left: proximal end-to-side anastomosis of the graft with the splenic vein. In the middle: great saphenous vein.

**Figure 2:**
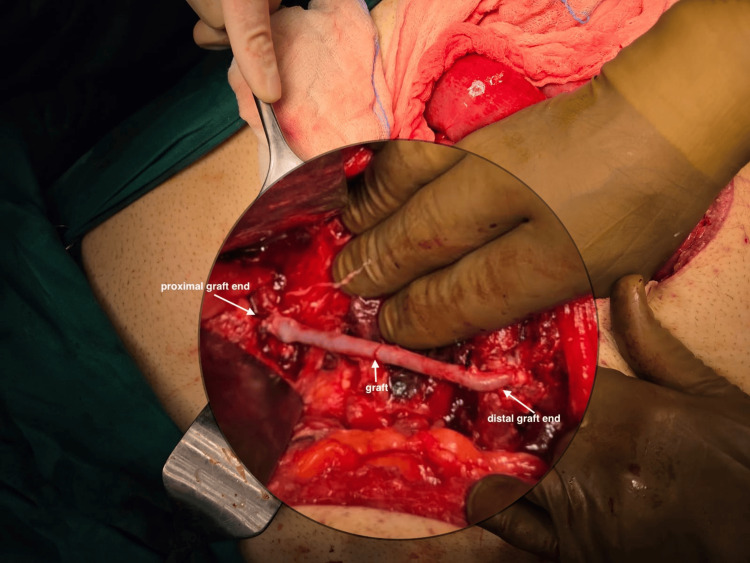
Magnified intraoperative image of the graft On the right: distal end-to-side anastomosis of the graft with a congested jejunal venous branch. On the left: proximal end-to-side anastomosis of the graft with the splenic vein. In the middle: great saphenous vein (graft).

Intraoperatively, the right colon and the right colonic flexure were mobilized uneventfully. However, due to excessive visceral adipose tissue and bulky mesentery, the ileocolic pedicle was not easily recognizable during the medial dissection. Thus, misperception of the vascular anatomy during high vascular ligation led to accidental ligation of a large, formal venous structure. The remaining part of the procedure was nearly complete, with the specimen having been resected. The OR team at that moment had to address a hemodynamically deteriorating patient with profound venous congestion of the entire small bowel. In the effort to assess the extent of vascular deficit and determine feasible options to restore the venous return of the small intestine, a vascular surgeon was consulted. Further dissection revealed a complete transection of the SMV, which was ligated at the level of its confluence with the splenic vein. The team decided to repair the injury by performing an autologous venovenous graft using the right great saphenous vein (GSV). Thus, the lateral aspect of a congested jejunal venous branch and the proximal splenic vein were used for the distal and proximal anastomosis of the graft, respectively. The vessels were anastomosed in an end-to-side fashion with continuous sewing using a 6-0 prolene suture (Figures 1, 2). Intraoperative Doppler sonography confirmed adequate blood flow through the graft and the portal vein. The entire operation was associated with less than 60 cc of blood loss, required no transfusion intraoperatively, and the net operative time was 270 minutes.

**Figure 3:**
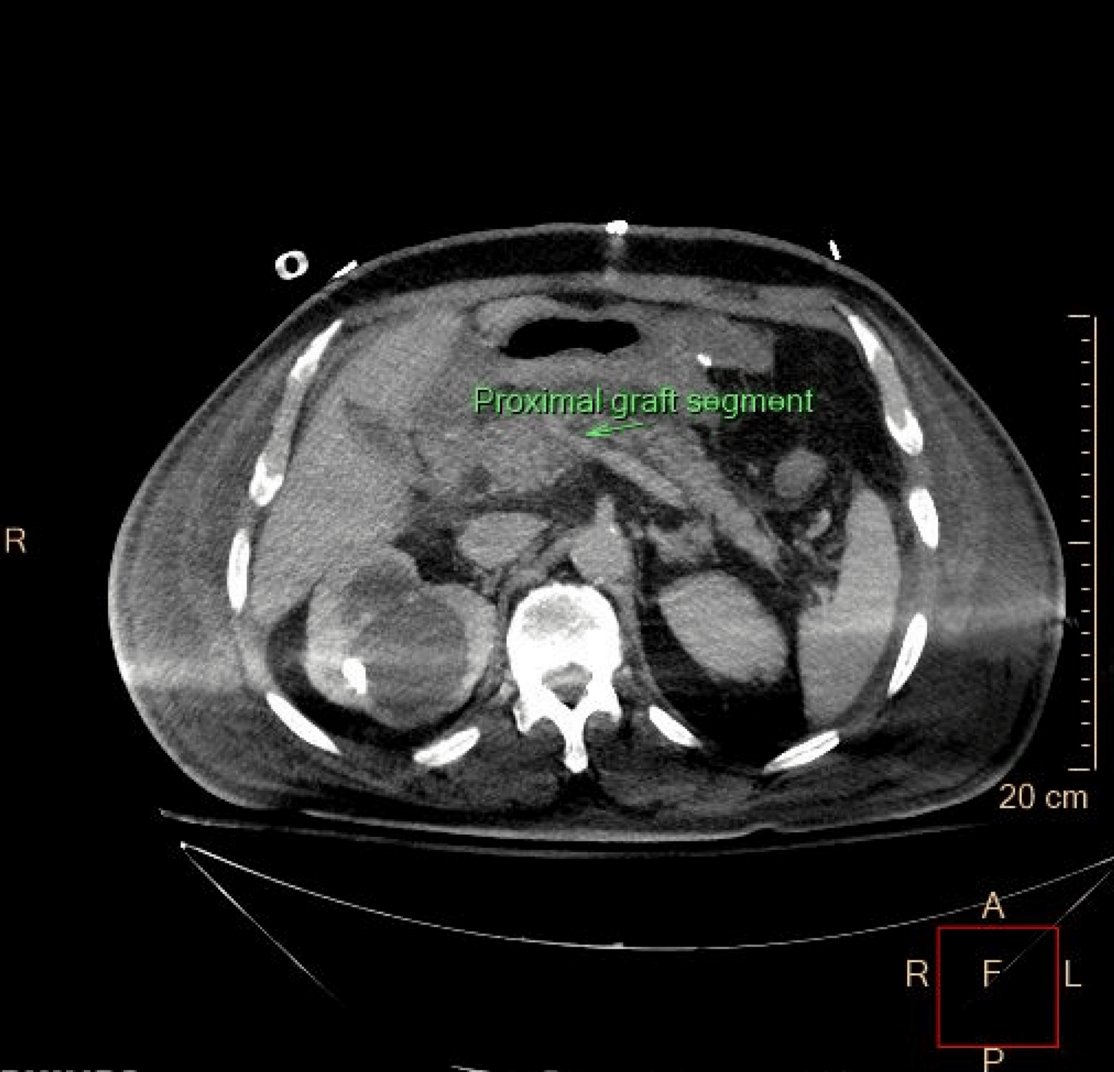
Postoperative CT scan confirming the patency of the graft The arrow shows the proximal end of the graft.

**Figure 4:**
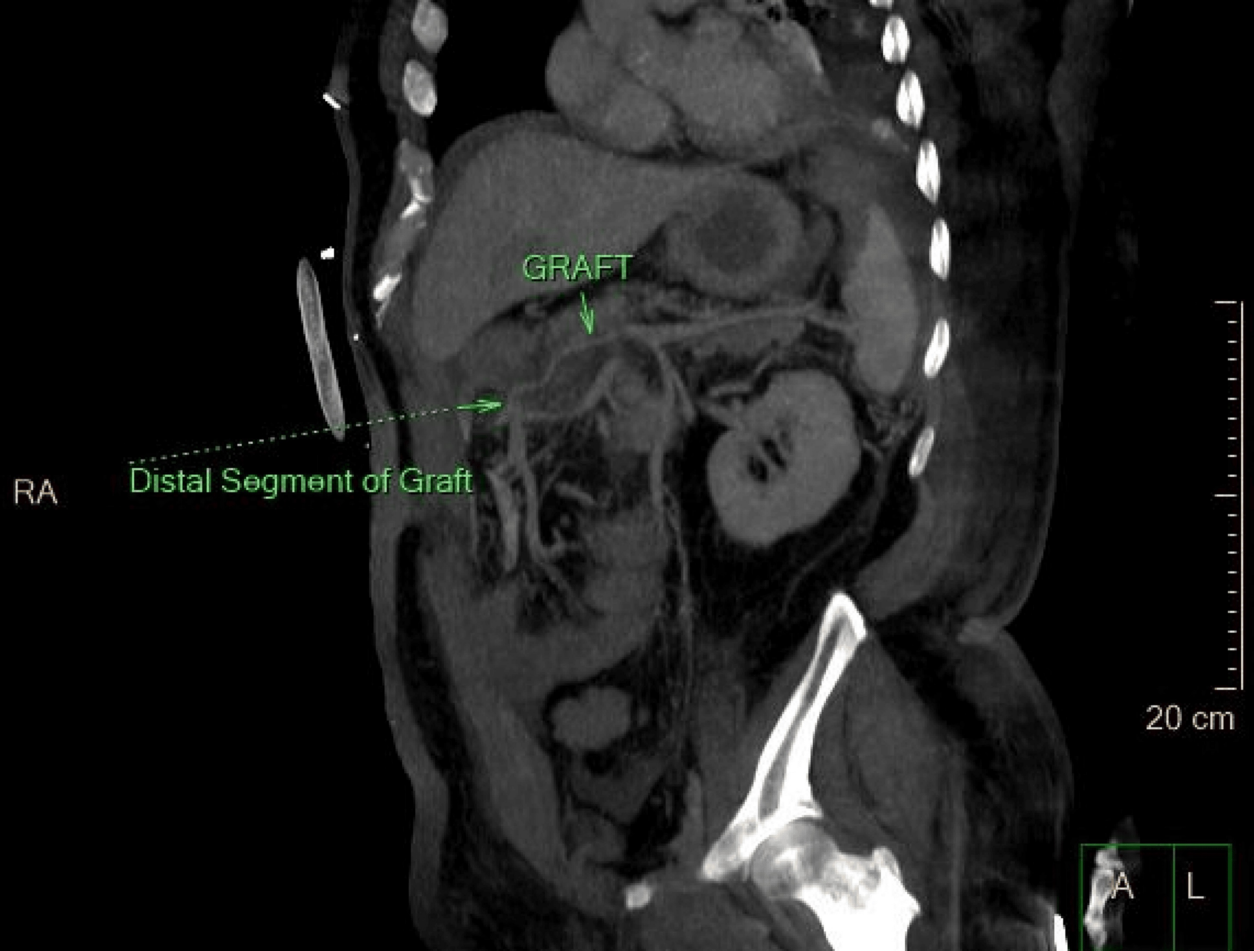
Postoperative image of the graft Sagittal view of the entire graft (right arrow) and its distal end (arrow with dotted line)

The patient was transferred to the surgical ICU with an end ileostomy, hemodynamically unstable, acidotic, and on two vasopressors. A new CT angiography was performed on postoperative day (POD) 4, given that his clinical condition improved, to assess the patency of the graft (Figures 3, 4). The patient was progressively weaned off pressors and extubated on POD 7. He received one unit of packed red blood cells during the ICU stay. He was transferred to the surgical floor on POD 9, alert, stable, tolerating enteral feeding, with a functional and satisfying output stoma. His hospital stay was prolonged due to a febrile, vancomycin-resistant enterococcal peritoneal fluid infection and *Acinetobacter baumannii* urinary tract infection, both of which were adequately managed with the appropriate antibiotics. The pathology report described a moderately differentiated, T4N2 colon adenocarcinoma. The patient was discharged home on POD 21, continuing on therapeutic oral anticoagulation with the plan for adjuvant chemotherapy and stoma take-down after a reasonable follow-up timeframe.

## Discussion

CME with high vascular ligation has been endorsed for its optimal oncologic outcomes. However, it comes with a pertinent price, which is the risk of potentially devastating injuries [10]. An iatrogenic SMV injury is a rare, yet potentially disastrous and quite underreported intraoperative adverse event during both laparoscopic and open right hemicolectomy with CME, which encompasses significant morbidity and mortality [11]. An SMV injury can vary in severity from a simple short-segment laceration due to avulsion, oversewing, or thermal injury to complete transection of a significant portion of the vessel. Common patterns of injury include excessive traction of the middle colic vein or the gastrocolic trunk of Henle, as well as misperception of vascular structures due to unrecognized anatomical variations [12]. Furthermore, patient characteristics merit mentioning. Bulky and fixed mesentery due to excessive adipose tissue can potentially lead the surgeon to incorrect dissection planes, increasing the risk of intraoperative injuries. There are numerous anatomical variations of the infrapancreatic vascular structures that should be taken into consideration by the surgeon who commits to performing radical oncologic colectomies [13]. Thus, colorectal surgeons should be quite familiar with the vascular anatomy of the area before being able to dissect the latter [14]. Preoperative multidetector CT angiography with 3D reconstruction has been advocated by several authors as a modality to map the anatomy of the vasculature along with its variations and, thereby, reduce intraoperative adverse events and enhance the surgeon’s confidence in the operative field [15,16]. Despite the existence of justified measures currently applied for the mitigation of the risk of potential vascular injuries, colorectal surgeons operating in high-volume centers are likely to face one in their career. Early recognition is paramount and relies on the experience of the surgical team as well as good communication between the surgeon and anesthesia team, so that deviations from normal physiology are detected as soon as possible. From this standpoint, few options of repair have been proposed for an iatrogenic SMV injury. Revascularization should be attempted whenever feasible. Primary venorrhaphy is a reasonable option for partial lacerations. In the context of complete transection without significant segment loss, primary end-to-end anastomosis, albeit rarely performed, can offer restoration of continuity in select cases. Autologous grafting using the GSV or the superficial femoral vein is the preferred mode of repair for injuries with significant segmental loss. In those circumstances, it is critical to avoid synthetic grafts due to the risk of contamination [17].

## Conclusions

An SMV injury is a rare and much underreported intraoperative complication during both minimally invasive and open right hemicolectomy. CME with high vascular ligation, despite the oncologic benefit, is associated with an increased risk of adverse events compared to the conventional approach. This report highlights that such complex and demanding cases would benefit most when managed by specialists in high-volume centers. Colorectal surgeons who are adequately familiar with the vascular anatomy of the field and follow standardized pre and intraoperative plans are more likely to mitigate the risk of intraoperative complications. Early recognition of any iatrogenic vascular injury is crucial, and all available resources and modalities of revascularization should be recruited as soon as possible.
